# Challenges of medical commodity availability in public and private health care facilities in the Upper East Region of Ghana: a patient-centered perspective

**DOI:** 10.1186/s12913-023-09717-9

**Published:** 2023-07-01

**Authors:** Oswald Atiga, Jackie Walters, Noleen Pisa

**Affiliations:** 1 Department of Transport and Supply Chain Management, University of Johannesburg, Auckland Park, South Africa; 2 Department of Procurement and Logistics Management of the Bolgatanga Technical University, Bolgatanga, Upper East Region Ghana

**Keywords:** Medical commodity availability, Public health care facilities, Private health care facilities, Patient-centered perspective, Upper East Region, Ghana

## Abstract

This article is a patient-centered comparison of medical ccommodities availability in public and private health care facilities in the Upper East (UER) of Ghana to determine if significant differences existed. A concurrent mixed method strategy was used where both quantitative and qualitative data were simultaneously collected, independently analysed and triangulated at the intepretation stage. Quantitative data were collected using a systematic sampling method where a total of 1500 patients (750 from public and 750 from private) health care facilities responded to the interviwer-administered questionnaires for this study. Exploratory factor analysis (EFA) was applied as a construct validation tool while a T-test was computed to compare if a significant difference existed between both type of patients. Qualitative data were collected from selected patients and heads of public and private healthcare facilities using an interview guide. The qualitative data were analysed using content analysis. The results indicated significant differences existed in the availability of medical commodities, frequency of medicine stock-outs, seasonality of medicine stock-outs, patients’ reaction to medicine stock-outs and communication about the medicine stock-outs to patients of private and public facilities. The biggest difference between the two groups of patients was how communication of medicines stock-outs was communicated to them.

Health care facility managers in the region must focus seriously on training staff on how to improve communication of medicines stock-outs to patients.

## Introduction

At the core of every healthcare delivery system is value-maximisation for patients by achieving the possible best health outcomes at the lowest possible cost. Patients are important consumers in the medical commodity supply chain as they are the end recipients/beneficiaries of the healthcare delivery system. Access to medicines and related medical commodities as and when needed, in any healthcare delivery system, depends on the availability, affordability and acceptability of such commodities [42].

The availability of medical commodities is a critical element to prevent, alleviate and cure infectious and preventable diseases [2]. The availability of medical commodities is a vital indicator for assessing the quality of any existing healthcare service delivery system [2] and is influenced by factors, such as the quality and capacity of drug storage facilities, availability of efficient transport and transport systems, the number of trained healthcare workers, inventory management practices and the finances to pay for such medical commodities [42].

Patients’ perceptions of service quality have become central in evaluating the competency, efficiency and reliability of any healthcare delivery system [7]. Such opinions would be based on the services rendered to patients by hospitals, clinics, health centres or community health planning service (CHPS) compounds. When the needs of patients are poorly handled by staff and the commodity delivery chain, it is expected to ultimately and negatively impact the healthcare facility’s competitive position [3]. Over the last 25 years, the role of the consumer (patient) has gained widespread recognition as a measure of quality of both private and public healthcare delivery services.

In Ghana, patients attending public healthcare facilities must be registered with the National Health Insurance Scheme (NHIS) in order to be able to access free health care, particularly outpatient services, doctor consultations, diagnoses and drugs specified under the scheme [6]. In the case of private facilities (private-for-profit), all costs associated with access to health care are paid by the patient, while in the case of private-not-for-profit facilities, the cost is subsidised, based on the facility’s policy or availability of funding sources [36].

### Statement of the problem

Several studies have investigated different aspects of patients’ experiences of healthcare facilities across sub-Saharan Africa (SSA). Such studies included last mile facilities not near to patients’ homesteads resulting in long distances to access medical care [37], as well as ineffective flow of commodities to last mile patients, limited transport for medicines to rural healthcare facilities [24] and disparities in the supply of essential mediciness to private and public patients in SSA [40]. Considering SSA’s portion of the world population, it is imperative that health care commodity supply chains are strengthened to effectively deliver medicines, medical equipment, medical technology, pharmaceutical products and related medical commodities. This is particularly significant for low-resource countries, especially in SSA, where access to essential medicines and related medical commodities are limited and irregular [28] and Kupfer, 2018). It is estimated that a third of the world’s population and more than half of the population (2 billion people) of low and middle-income countries (LMICs), particularly in SSA, lack access to medicines and related medical commodities, diagnostic equipment and other health technologies [1]. The paucity of regular supply of medical commodities comes against the backdrop of SSA contending with the highest portion of the world’s disease burden [16]. Despite the various studies, no patient-centered comparative study has compared the medical ccommodities availability in public and private health care facilities in the Upper East (UER) of Ghana for which this study has done.

### Main objective of the study

The overall objective of the study is.

i. To conduct a patient-centered comparison of the challenges of medical commodities availability in public and private health care facilities in the UER of Ghana.

Other objectives of the study are.

i. To determine if differences exist in the challenges affecting the medical commodities availability in public and private health care facilities in the UER of Ghana.
ii. To calculate the effect size if significant differences exist.

## Literature review

This section of the study reviews the extant literature. “Theoretical review” section presents the theoretical review whilst “Empirical review” section continues with the empirical review.

### Theoretical review

#### Andersen’s model of health service use

Andersen’s Model of health service use (Andersen 1995) was developed to study the factors that determined one’s health services utilization, assess the inequalities encountered in accessing health care services and facilitating the policy-making processes for equitable access to health care services [8]. This Model was originally proposed by Andersen (1968) and subsequently modified by Andersen and Newman (1973) culminating in a theoretical framework seeking to understand and explain the determinants and reasons people preferred and used certain types of health services or general types of health services. The Model argued and concluded that people’s use of health services is a function of their predisposition. Andersen’s original behavioural model has continuously undergone amendments over the years with renewed focus on such factors as ‘consumer satisfaction’ in the 1970s and ‘health status’, ‘personal health practice’ and ‘external environment’ in the 1980s. Andersen made a personal review of the model in 1995 and introduced feedback loops into the framework.

[33] applied the Andersen-Newman model of health care utilization to understand antenatal care use in the Kersa District of Eastern Ethiopia. The study concluded that more than half of the women (respondents) attended at least one antenatal care visit while a sizable proportion of the women had infrequent and delayed antenatal care attendance. Measures to improve antenatal care utilization among women in the Kersa district included improving peer education programs to mobilizing and supporting their educational achievement, pursuing programs to change husbands’ attitudes, ameliorating the quality of antenatal care, increasing the Health Extension Worker’s home visits program and increasing the awareness of pregnancy complications [17] also applied Andersen’s behavior model to identify factors influencing maternal healthcare service utilization in Bangladesh. The study concluded that universal education and diminishing geographical disparities of healthcare access were some of the factors that influenced the desirable use of maternal healthcare services in Bangladesh. Another study using Andersen’s behavioral model of health care utilization in a decentralized program to examine the use of antenatal care in rural western Ethiopia was conducted by [35]. The study concluded that birth size and spacing, knowledge of pregnancy complications and benefits of minimum ANC visits (antenatal clinic visits), local socio-economic development measures targeting poor women/households and further decentralization of health system and improving proximity to ANC in rural western Ethiopia were factors to consider in health care facilities utilization. This study is a patient-centrered comparative study of the challenges of medical commodity availability in private and public health care facilities in the UER of Ghana.

### Empirical review

#### The state of Ghana’s healthcare delivery system

Ghana is a West African country with ten political regions (as at the time of this study) divided into 216 metropolitan, municipal and district assemblies [25]. The country has a population of 30.8 million, an increase from the official 2010 census figure of which 50.9% is male and 49.1% female [15]. Like most sub-Saharan African (SSA) countries, where almost 75% of deaths occur from non-communicable diseases, Ghana has a high disease burden with a significant rise in communicable and non-communicable diseases in recent years [20]. Ghana’s healthcare delivery capacity has been weakened by an increase in malaria, tuberculosis, HIV/AIDS and other diseases [41], such as diarrhoea and lower respiratory infectious diseases, accounting for almost 50% of all deaths in Ghana [13]. Malaria particularly, is a significant cause of adult sickness and deaths, and the leading cause of absenteeism among workers and school-going children [27]. This disease is endemic and accounted for 40 to 44% of out-patients, 22% of under-five-year-old child mortality, 13% of hospital deaths and 35% of all admissions to healthcare facilities across Ghana [10].

To mitigate the actual and net effect of Ghana’s huge disease burden and the general weaknesses in the healthcare system, requires a systematic strategy to strengthen both the private and public medical commodity supply chains.. Ghana’s national drug policy is to improve and sustain the health of Ghanaians by emphasising the rational use and access to safe, effective, good quality and affordable medicines [13]. This policy places issues of accessibility, availability, affordability and acceptability of medical commodities to patients at the forefront of healthcare delivery.

In Ghana, the last mile delivery points for medical commodities are district clinics, healthcare centres and community-based healthcare planning system (CHPS) compounds in the rural and far-to-reach areas. These facility-types are the final destination of Ghana’s public healthcare delivery system in the most remote parts of the rural communities; these at the district, sub-district and community levels are the last mile facilities. CHPS compounds, just like drug stores, deliver basic medical services and medical commodities to rural patients.

The UER is located in the North-Eastern corner of Ghana. It borders the republics of Burkina Faso to the north and Togo to the east [25]. The region has 13 administrative districts and municipalities that is, ten districts and three municipalities as at the time this study was conducted [25]. With a population of 1.3 million, the region has one of the poorest healthcare infrastructures and amenities in the country [15]. There are 233 CHPS compounds, 48 clinics, 48 district hospitals, 44 healthcare centres and one regional hospital [14]. Other challenges in the region, include patients walking long distances to access healthcare facilities [37], the lack of adequate transport, the perennial outbreak of meningitis, poor and inaccessible roads, particularly in the rural areas and during rainy seasons, and a limited number of health professionals across healthcare facilities [14].

There is low availability of medical commodities, particularly in the public sector of low and middle-income countries (LMICs), compared to the private sector [31]. On average, the availability of medical commodities in LMICs is 35% in public facilities and 66% in the private sector, although prices are often high and unaffordable in the latter [2]. In Ghana, the availability of medical commodities, especially in the rural and last mile rural areas, are often in short supply [6]. Shortages of antiretroviral drugs were reported from all healthcare facilities, making access to these medicines problematic [22]. A World Bank report on the state of healthcare delivery in Ghana, concluded there were shortages of drugs and medical equipment, especially at lower-level healthcare delivery facilities [43]. A report on the shortage of medicines within the public healthcare supply chain, showed the average percentage of days was greater than 50% for out-of-stock of 20 tracer drugs in both the regional and central medical stores [5]. A holistic assessment of the health sector programme for Ghana’s Ministry of Health (MoH) identified challenges, such as inadequate funds for community activities, inadequate human resources and the perennial shortage of psychotropic medicines [14].

Reasons for shortages of medical commodities in Ghana are due partly to inefficiencies within the private and public health commodity supply chains. Such inefficiencies, include poor co-ordination among supply stakeholders, inadequate means of transport, poor road infrastructure and poor procurement practices, resulting in higher operational and distribution costs and higher medicine prices, especially at the last mile level [21, 26]. The lack of medicines and related medical commodities undermines the ability of the healthcare professionals to respond to patients’ needs, a situation that has the potential of eroding patients’ confidence and trust, not only in the professionals, but in the entire healthcare system [4].

## Research process

“Research process” section presents the research design, including the qualitative and quantitative approaches to the study. This was a mixed-method research design that combined elements of quantitative and qualitative research techniques into a single study. The qualitative and quantitative phases of the study were concurrently conducted, the results separately analysed, then combined in the reporting stage to confirm the perceptions of the availability of medical commodities for the patients of private and public healthcare facilities in the UER of Ghana.

### Population and sample size

Since the population of patients attending the four types of study health care facilities (health care centres, clinics, hospitals and CHPS compounds) in the region was unknown, the researchers conducted a pilot study to determine the average number of patients who attended these facilities. The pilot study involved patients of facilities of four (4) non-study districts – the Pusiga, the Builsa South, the Bongo and Tongo districts. The results of the pilot test indicated on average between 25 and 35 patients attended these facilities on a daily basis. Whilst the smaller and hard-to-reach facilities recorded lower than 30 patients, the relatively bigger facilities attracted slightly above 35 patients per day. Based on these results, the researchers settled on 30 patients per facility as the average daily attendance for patients attending health care in the region since 30 was the average of 25 and 35. This therefore meant 30 patients per facility × 25 facilities equals750 patients for the public and same for the private health care facilities resulting in a total of 1500 respondents for this study. These 750 patients each were drawn from the 50 health care study facilities (25 public and 25 private) involved in the study. The use of large sample size has been used in this study because analytical tools such as the EFA, the t-test have encouraged the use of large sample sizes especially for causal-comparative, experimental-type research, or surveys, where inferential statistics need to be calculated [9, 30]. The study by [29] provided a justification for using a large sample size. It indicated that “the adequacy of sample size in an EFA might be evaluated very roughly on the following scale: 50-very poor, 100-poor, 200-fair, 300-good,500-very good, and 1000 or more-excellent.” Aside being more representative of the population while limiting the effects or influence of outliers in a particular study, large sample size studies are generally more reliable [30]. One other important justification for adopting large sample size is its precision when it comes to the calculation of effect size. It also allows for an easier assessment of the representativeness of the sample and the generalisability of the research findings [30].

### Data collection

This study adopted a mixed methods strategy using quantitative and qualitative techniques. The qualitative data were collected from selected officials of the medical commodity supply chains, including the regional director of health services (RHD), the manager of regional medical stores (RMS) and selected heads of private and public healthcare facilities. These participants were selected using a purposive sampling technique as they were considered knowledgeable and experienced to participate in the study. Qualitative data were collected using a semi-structured interview guide.

The systematic sampling method was used to collect the quantitative data from 30 patients each from 25 selected public and private health care facilities making 750 patients for the public and 750 for the private health care facilities making up 1500 patients in total for this study from a target population of 1980. Every second patient who had previously attended a healthcare facility and was willing to participate in the study was given a questionnaire to complete. For a respondent (patient) to be eligible to complete a questionnaire, they were required to have accessed healthcare services at least twice within the past two years. This was to ensure that patients had some experience to have an informed opinion of a particular facility’s services. There were eight sections in the questionnaire: “Introduction” section included questions on the location of the facility (district) and if the facility was public or private. “Literature review” section asked patients if they were registered members of the NHIS or not, Sections 3 to 6 enquired of the respondents (patients) their perceptions of the following factors: availability of medical commodities, reaction to commodity non-availability, causes of medical commodity stock-outs, and incidences of commodity non-availability. Other questions included the effects of seasonality on commodity non-availability and communication about stock-out information. Apart from the ‘yes’ or ‘no’ questions, the questionnaire had a five-point Likert-scale with the variables of strongly disagree; disagree; neutral; agree; strongly agree, and single response questions; don’t know; never; rarely; occasionally; frequently; always.

Thirty (30) patient questionnaires were pilot-tested using systematic sampling among selected patients of four healthcare delivery facilities (two public, two private) within the Bongo district, a non-study district. This pilot test was carried out in the Bongo district hospital (public facility), the St. Theresa’s Health Centre (private-not-for-profit CHAG) and the AYIRE Clinic (private), a newly constructed facility. Every third patient who came into each of these facilities seeking medical attention, participated in the pilot test. The outcome of the pilot test resulted in adjustments to certain questions. Some of the amendments to the questions involved rephrasing, others were removed. The pilot-testing resulted in the number of questions being reduced from 17 to 14 in the final questionnaire. Of the total population of 1980, data was collected from 1500 patients from both public and private health care facilities during the three month period of data collection*.*

For the qualitative data, there was an interview guide consisting of two (2) sections. The first section contained bio-data questions of patients such as gender, age, type of health care facility attended, number of times patient attended facility. The second section of interview guide posed a total of eleven (11) eleven open-ended questions which ranged from medical commodity availability, communication about stock-out information, causes of medical commodity stock-outs, incidences of commodity non-availability, reaction to commodity non-availability and effects of seasonality on commodity non-availability in health care facilities in the region.

### Data analysis

The qualitative data were analysed using content analysis where data were transcribed, coded and key thematic areas identified [32]. The written version of the recorded interviews was transcribed for the purposes of analysis. The transcribed data were reduced to smaller common themes to identify and explore relationships among the variables. The coding was ‘by hand’, using coloured pencils to denote certain thematic areas.

The quantitative data were analysed as follows: i) exploratory factor analysis (EFA) computed as a construct validation tool identifying the latent construct scales, ii) descriptive statistics, namely means and standard deviations determined the magnitude of each factor influencing the efficiency of the medical commodity chains, iii) t-tests were computed to compare if a statistical significance existed between the patients of private and public healthcare facilities regarding medical commodity stock-outs in the region, iv) effect sizes were computed to determine the significance of the differences between patients of private and public healthcare facilities in the region (see Tables 7, 8, 9, 10 and 11).

Since this study adopted a mixed methods strategy, the triangulated results are discussed taking the literature, the interviews and survey results into consideration. The results of this study are presented in “Results” section.

## Results

This section of the study presents the analysis of the results.

### Number of patients in the study

The number of questionnaires completed were 1500. Of these, 51% of the respondents attended public healthcare facilities, whilst 49% sought health care in private facilities.

### Types of medicines with high frequency of stock-outs

The study compared frequently out-of-stock medical commodities between private and public healthcare facilities. The results in Table 1 indicate that 43.2% of those using private healthcare facilities and 48.3% of patients of the public healthcare facilities agreed/strongly agreed that they experienced frequent stock-outs of anti-malaria drugs; only 29.1% of private patients and 56.4% of those using public facilities agreed/strongly agreed, experienced frequent stock-outs of contraceptives. The highest frequency of stock-outs of vaccines was agreed/strongly agreed by 54.9% of private and 53.7% of public facility patients. Most patients from both types of facilities, private 56.8% and public 55.6% agreed/strongly agreed that they did not always receive all their required essential commodities, due to the high rates of stock-outs.

**Table 1:** Types of medicines with high frequency of stock-outs

**Patients’ experiences of non-available commodities in health facilities**	**Private facility patients**			**Public facility patients**		
	**strongly disagree/disagree**	**neutral**	**strongly agree/agree**	**strongly disagree/disagree**	**neutral**	**Strongly agree/agree**
Experience incidence of stock outs of anti-malarias whenever I visit this facility	40.7%	16.1%	43.2%	34.7%	17.0%	48.3%
Experience incidences of stock outs of contraceptives whenever I visit this facility	41.4%	29.5%	29.1%	23.6%	20.0%	56.4.%
Experience incidences of stock outs of vaccines whenever I visit this facility	35.3%	9.8%	54.9%	27.6%	18.7%	53.7%
Do not always get all my required medical commodities due to high stock out rate	18.5%	24.7%	56.8%	16.2%	28.2%	55.6%

Source: Author’s own compilation (2021)

The interview results from the Upper East Regional Director of the Ghana Health Service [RD-UER] supported the findings:

W*e [GHS] recognise the sporadic unavailability of commodities in some of our facilities but you would also acknowledge the pressures on our facilities by the ever-increasing demands posed by the NHIS coupled with weak internally generated funds*... [RD-UER, 2018].

### Frequency of medicine stock-outs

**Fig. 1:**
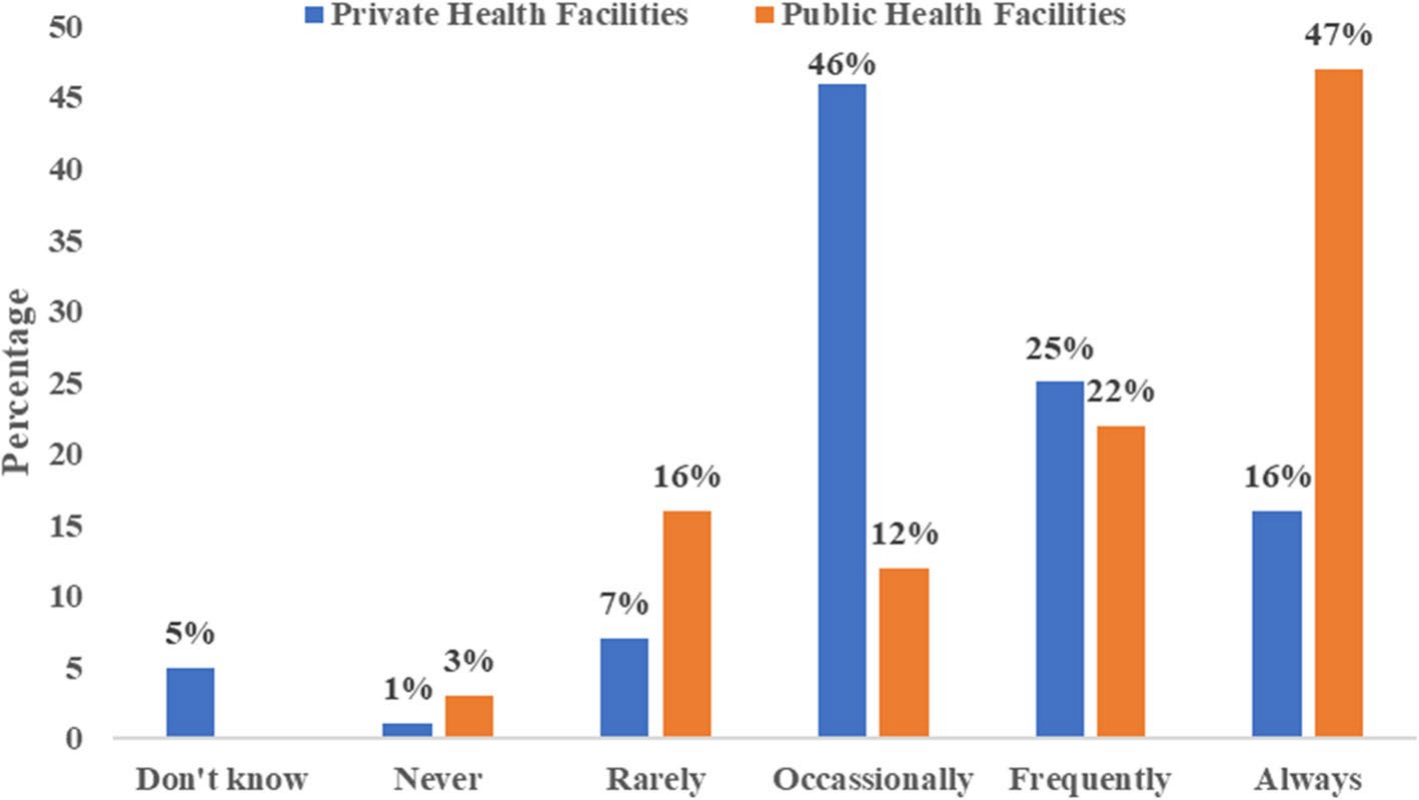
Frequency of medicine stock-outs across health care facilities. Source: Author’s own compilation (2021)

It is the expectation of every patient to access good quality medicines from an efficient healthcare system. The results indicate that the majority (69%) of patients of public health care facilities always (47%) or frequently (22%) did not receive commodities during visits (see Fig. 1). For patients of private facilities, the results were that 46% occasionally did not receive commodities during routine visits. The results demonstrate a high frequency of non-receipt of commodities in both public and private healthcare facilities in the UER.

### Exploratory factor analysis (EFA)

Exploratory factor analysis (EFA) was applied as a construct validating technique to authenticate the credibility of the data collected from the patients’ questionnaires. The Kaiser–Meyer–Olkin (KMO) test for sampling adequacy and Bartlett’s test of sphericity determined the suitability of the data for factor analysis. Bartlett's test of sphericity tests that the correlation matrix, which is an identity matrix, would indicate that the variables are unrelated and therefore, unsuitable for structure detection. The validated results in Table 2 showed a KMO test statistic of 0.755 and a statistically significant value (*p-value* $<$ *0.000*) for the Bartlett's test of sphericity indicating the data were suitable for factor analysis. A factor is included if its loading is greater than 0.5.

**Table 2:** KMO and Bartlett’s test

Kaiser–Meyer–Olkin measure of sampling adequacy	.755
Bartlett’s Test of Sphericity	
Approx. Chi-Square	7271.194
Df	136
Sig	.000

Source: Author’s own compilation (2021)

Five factors, namely i) availability of medical commodities (AMC), ii) frequency of medicine stock-outs (FMS), iii) seasonality of medicine stock-outs (SMS), iv) reaction to medicine stock-outs (RMS) and v) communication about the medicine stock-outs (CMS) were identified by the EFA (Table 3). In addition, a reliability analysis was conducted of the patients’ survey instrument to assess the internal consistency of the variables. The results showed reliability coefficient (Cronbach’s alpha) values ranging from 0.863 for the availability of commodities, 0.772—frequency of medicine stock-outs, 0.703—seasonality on medicine stock-outs, 0.504—reaction to medicine stock-outs, and 0.770 for communication about the medicine stock-outs to patients, indicating a strong internal consistency among the variables, thus suggesting the suitability of the data for EFA.

**Table 3:** Reliability of factors

	**Cronbach’s Alpha**
Availability of commodities (AMC)	.863
Frequency of medicine stock-outs (FMS)	.772
Seasonality of medicine stock-outs (SMS)	.703
Reaction to medicine stock-outs’ (RMS)	.504
Communication about the medicine stock-outs to patients (CMS)	.770

Source: Author’s own compilation (2021)

### Loaded factors

The EFA output shown in Table 4 explains the total variance of both the loaded and unloaded factors. A total of five factors with Eigen values greater than 1 explained 63.91% of the total variance. Factor 1 explained 18.59%, factor 2—16.76%, factor 3—13.27%, factor 4—8.40%, while factor 5 explained 6.89% of the total variance. The grouping of these factors with their underlying items are presented in Table 4.

**Table 4:** Total variance explained

Component	Initial eigenvalues			Extraction sums of squared loadings			Rotation sums of squared loadings		
	Total	% of Variance	Cumulative %	Total	% of Variance	Cumulative %	Total	% of Variance	Cumulative %
1	3.161	18.592	18.592	3.161	18.592	18.592	2.946	17.330	17.330
2	2.849	16.761	35.353	2.849	16.761	35.353	2.477	14.570	31.900
3	2.256	13.270	48.623	2.256	13.270	48.623	2.395	14.090	45.990
4	1.427	8.397	57.020	1.427	8.397	57.020	1.611	9.474	55.464
5	1.171	6.889	63.909	1.171	6.889	63.909	1.436	8.445	63.909
6	.873	5.138	69.047						
7	.806	4.743	73.790						
8	.661	3.889	77.679						
9	.637	3.748	81.427						
10	.526	3.093	84.520						
11	.503	2.958	87.478						
12	.475	2.794	90.272						
13	.424	2.493	92.764						
14	.378	2.224	94.988						
15	.343	2.016	97.005						
16	.279	1.644	98.648						
17	.230	1.352	100.000						

*Note*: Extraction method: Principal Component Analysis

Source: Author’s own compilation (2021)

The results in the suppressed rotated exploratory factor analysis matrix (Table 5) indicate the loaded factors with eigen values greater than 1. The first loaded factor is named i) availability of medical commodities (AMC), ii) the second factor is frequency of medicine stock-outs (FMS), iii) the third factor is seasonality of medicine stock-outs (SMS), iv) the fourth factor is reaction to medicine stock-outs (RMS) and v) the final factor is communication about medicine stock-outs to patients (CMS).

**Table 5:** Suppressed rotated exploratory factor analysis and reliability analysis

	**Component**									
	**1**	**2**	**3**	**4**	**5**	**KMO**	**Bartlett’s Test of Sig**	**Eigen Values**	**% of Variance**	**Reliability**
AMC1	**.881**	-.076	.068	-.094	.125					
AMC4	**.877**	-.049	-.064	-.027	.152					
AMC2	**.770**	.005	-.328	.126	-.010			**3.161**	**18.592**	**0.863**
AMC3	**.767**	-.109	.222	-.050	-.016					
FCS 3	-.037	**.826**	.075	.012	.076					
FCS 2	.061	**.795**	.227	-.097	.108					
FCS 1	-.155	**.750**	.050	.010	-.020			**2.849**	**16.761**	**0.772**
FCS 4	-.059	**.664**	.021	.217	-.044					
SCS 4	.038	.035	**.790**	-.024	-.092	**0.775**	**0.000**			
SCS 1	-.104	.146	**.751**	-.165	.101			**2.256**	**13.270**	**0.703**
SCS 3	.254	.185	**.668**	-.090	-.070					
SCS 2	-.152	.028	**.614**	.072	.166					
RCS2	-.001	.004	.089	**.770**	-.011					
RCS1	-.102	.011	-.179	**.691**	.296			**1.427**	**8.397**	**0.504**
RCS4	.022	.092	-.091	**.612**	-.108					
CMS 3	.047	-.079	.266	-.082	**.832**			**1.171**	**6.889**	**0.770**
CMS 2	.272	.259	-.197	.176	**.730**					
**Total Explained Variance**									**63.909**	

Rotation Method: Varimax with Kaiser Normalisation, Extraction method. *PCA* Rotation converged in 5 iterations

Source: Author’s own compilation (2021)

### Comparing factors affecting commodity availability between patients of private and public healthcare facilities

T-tests were computed to compare the factors affecting the commodity availability between patients of private and public healthcare facilities. The t-test analysis is presented with the following factor patterns; i) availability of commodities (AMC), ii) frequency of medicine stock-outs (IMS), iii) effect of seasonality factors on medicine stock-outs (SMS), iv) reaction to medicine stock-outs (RMS) and v) communication about the medicine stock-outs to patients (CMS).

### Availability of medical commodities

Table 6 shows the mean scores and standard deviation (SD) for patients of public (mean *2.73*) and private (mean *2.99*) healthcare facilities, indicating a higher availability in private facilities. The t-test results (*p-value* $<$ 0.000) indicate that statistically significant differences exist between private and public healthcare facilities regarding the availability of medical commodities. The survey results are supported by the results of the in-depth interviews, as shown in the highlights from the regional director of the Ghana Health Service below:

*… commodities are definitely more available in private compared to public health facilities in this region … sale agents of pharmaceutical manufacturing companies are available in their branded minivans and ready to supply as and when they are requested to. Whilst public facilities cannot just buy at will due to public procurement requirements, private care facilities can buy medicines as and when needed … . a reason for which commodity availability will always be higher in private facilities … … ..* [RD-UER, 2018].

**Table 6:** Effect size of the mean differences in the availability of commodities

	**Private facilities**	**Public facilities**	**ETA squared**	**Interpretation**
	*Mean (SD)*	*Mean (SD)*		
Availability of medical commodities	2.99 (0.95)	2.73 (1.01)	.017	Small effect

Source: Author’s own compilation (2021)

Both sets of findings are corroborated by findings from [39], who confirmed higher commodity availability for private healthcare facilities compared to those in the public low and middle-income countries (LMICs).

Since significant differences exist between the perceptions of patients of private and public healthcare facilities regarding the availability of commodities, the magnitude of the difference using the ETA squared (η^2^) statistic was subsequently inferred. In Table 6, the ETA squared value of 0.017 indicates a small difference between the patients of private and public healthcare facilities of the availability of medical commodities based on [9]’s (1988)^1^ guidelines for effect size. This could be that the patients of public healthcare facilities buy their non-available medicines from private pharmacies and drugstores. 

### Frequency of stock outs of essential medicines

Essential medicines satisfy the priority healthcare needs of a particular population. The basis for determining and selecting essential medicines in developing countries is directly related to the disease treated, their efficacy and safety, and their cost-effectiveness**.** Regarding the frequency of medicine stock-outs, the patients of public facilities experienced a higher frequency of essential medicine stock-outs (mean 3.27) compared to patients of private healthcare facilities (mean 3.11). The t-test results (*p-value* $<$ 0.000) indicate significant differences between patients of private and public healthcare facilities related to the frequency of stock-outs of essential medicines.

The survey results above are confirmed by the results of the in-depth interviews with patients from both types of health care facilities in the region:

A patient from a private healthcare facility stated the following:

*… I [patient of Private facility - PPrvtF] have “tasted” both types of health care facilities in this region as a patient and I can say that I experience better access to medical commodities anytime I attend a private health care facility … “Once I have the money to pay, I get all my medical commodity requirement from the private health care facility” Private facilities quickly restock their commodities the moment stocks go low … …* [*patients of Private facilities - PPrvtF*, 2018].

A patient from a public healthcare facility stated:

*… .I [patient of Public facility - PPuF*] have experienced high incidence of medical commodity stock-outs in *public health care facilities in this region.* Most often, when I attend this public facility for service I do not always get all my consignment of medicines *…* [*patient of Public facility - PPuF*, 2018].

Since there was a significant difference between the perceptions of patients of private and public healthcare facilities regarding the incidence of medicine stock-outs, there was a need to calculate the magnitude of the difference using the ETA squared (η^2^) statistic. As shown in Table 7, the ETA squared value of 0.011 implies that the effect size for the frequency of medicine stock-outs is small. Since essential medicines by their nature are in high demand, private or public healthcare facilities should make every effort to ensure their patients are provided with the right medications even if it means substituting drugs.

**Table 7:** Effect size for frequency medicine stock-outs

	**Private facilities**	**Public facilities**	**ETA squared**	**Interpretation**
	*Mean (SD)*	*Mean (SD)*		
Incidence of medicine stock-outs	3.11 (0.81)	3.27 (0.82)	.011	Small effect

Source: Author’s own compilation (2021)

### Effect of seasonality factors on medicine stock-out of commodities

Seasonality in some SSA countries is an influencing factor for certain medicines. The mean score for patients of private healthcare facilities for the seasonality of non-available commodities is relatively lower (mean 2.90) compared to the mean scores for public healthcare facilities (mean 3.11). The t*-*test results (*p-value* $\le$ 0.006) also indicate a significant difference exists in the effects of the seasonality of non-available commodities between the patients of private and public healthcare facilities. The results indicate public healthcare facilities are more prone to seasonality effects of medicine stock-outs. The results of the in-depth interview with the regional director of the Ghana Health Service support this finding, as indicated below:

*...as indigenes of this region we [patients of Public facilities – PpuF] … .are aware that May to somewhere September every year is the Malaria season because of the wet season … we [patient of Public facility – PpuF] … therefore expect that the health authorities would know these patterns and act appropriately to ensure more antimalarials are stocked up in order to prevent or at least minimise stock-outs during such periods … .. …* [*patient of Public facility - PPuF*, 2018].

*...we* [*patients of Public facilities - PPrvtF*, 2018] know Ghana and for that matter the Upper East Region (UER) is a Malaria endemic region and *as such private health care facilities usually receive high numbers of patients during the rainy season – May to September each year. We therefore expect managers of these facilities to fully stock-up before the season … …* [*patient of Public facility - PPrvtF*, 2018].

The qualitative and quantitative results are corroborated by [23], who established seasonality as a critical factor in commodity-demand in Zambia [23] asserted that seasonality patterns influenced the demand for malaria products, not only in Zambia but across SSA. The significant difference between the perceptions of patients of private and public healthcare facilities regarding seasonality factors of medicine stock-outs, reflects a need to compute the magnitude of the difference. Comparing the calculated ETA squared value of 0.019 (Table 8) with the commonly used guidelines proposed by [9], i.e., 0.01 small, 0.06 moderate, 0.14 large effect, implies that the effect size for the seasonality of medicine stock-outs is small. This small difference could be that all healthcare facilities (private and public) in the region are affected by the same season.

**Table 8:** Effect size for seasonality factors on medicine stock-outs

	**Private Facilities**	**Public facilities**	**ETA squared**	**Interpretation**
	*Mean (SD)*	*Mean (SD)*		
Effect of seasonality factors on medicine stock-outs	2.90 (0.80)	3.11 (0.75)	.019	Small effect

Source: Author’s own compilation (2021)

### Reaction to medicine stock-outs

Patients react differently when prescribed medicines (stock outs) are not available at healthcare facilities; the mean scores for patients of private (mean 1.71) and public facilities (mean 1.78) were found to be low for both (Table 9). The t-test results (*p-value* $\le$ 0.006) also indicated significant differences existed in the reaction to medicine stock-outs between the patients of private and public healthcare facilities.

**Table 9:** Effect size of mean differences in reactions to medicine stock-outs

	**Private facilities**	**Public facilities**	**ETA squared**	**Interpretation**
	*Mean (SD)*	*Mean (SD)*		
Reaction to medicine stock-outs	1.71 (0.43)	1.78 (0.62)	.005	Small effect

Source: Author’s own compilation (2021)

Highlights of the in-depth interviews involving the patients of private and public healthcare facilities support the survey results. In the words of the patients of public facilities,

*.......we [patients of Public facilities - PPuF] … ”know automatically we have to buy medicines from private drugstores and pharmacies whenever stock-outs of medicines occur from the public health care facilities we attend.. … the next thing is that we receive prescriptions forms to go and buy these medicines from external sources … .”* [PPuF 1, 2018].

In the words of a patient of a private health care facility,

*because these facilities are private.. … we [patients of Private facilities - PPrvF] know that even in times of stock-out, some substitutes drugs (whether good or poor) will be made available by these facilities for us to buy … so we tend to spend more money.. … sometimes on very expensive substitute drugs whose efficacy we are often not too sure of..* [PPrvF - 1, 2018].

The magnitude of the difference of the patients` reactions to medicine stock-outs was found to be 0.005 (Table 10), which is a small effect compared to the [9] (1988) guidelines. There is a small difference in the reaction between patients of private and public healthcare facilities because medicine stock-outs affect all patients. Private healthcare facilities operate purely on a profit basis, they, therefore, often tend to prescribe substitute drugs for their patients.

**Table 10:** Effect size of mean differences in communication of the medicine stock-outs

	**Private facilities**	**Public facilities**	**ETA squared**	**Interpretation**
	*Mean (SD)*	*Mean (SD)*		
Communication about the stock-out information	2.60 (0.96)	3.24 (0.98)	.100	Fairly large

Source: Author’s own compilation (2021)

### Communication about the medicine stock-outs to patients

The need for communication about the commodity availability to patients is critical to ensure they are adequately informed, especially of the reasons for medicine stock-outs. The mean scores for communication about the medicine stock-outs to patients of private (mean 2.60) and public (mean 3.24) healthcare facilities are presented in Table 10. These results indicate better levels of communication about the medicine stock-outs to patients in public than in private healthcare facilities. The t-test results (*p-value* $<$ 0.000) also indicate a statistically significant difference. Patients of both a private and a public healthcare facilities confirmed this finding, as indicated in the respective interview highlights shown below:

*.....communication of stock-out information in the public health care facilities is done in a better fashion by the staff compared to their counterparts in the private health care facilities … ..in the case of public facilities the issuance of a prescription form is the first indication of stock-out of one drug or the other. This is followed by an explanation from the concerned health staff to us [patients of Public facilities - PPuF] regarding the circumstances of the stock-out and an indication of where to go and buy the rest of these medicines … in the case of private facilities, the issuance of a prescription form should be enough information for us [patients of Private facilities - PPrvF] to understand that there are stock-out of some drug or the other … .the only other thing private health facilities do better is going the extra mile to prescribe poor substitute drugs for us [patients of Private facilities - PPrvF]-* [PPrvtF and PPuF 1, 2018].

The interview excerpts above indicate the compared perceptions of patients of private and public healthcare facilities on the communication of stock-outs information to patients in the region. The excerpts indicate better communication of stock-out information from public health care facilities compared to private health care facilities.

The effect size for communication about the medicine stock-outs to patients is comparatively larger (0.1) (Table 10). The possible reason for this significantly better communication of stock-out information in public health care facilities compared to private facilities to patients could be due to the improved training or/and capacity building workshops that the GHS has been periodically organizing for the staff of public healthcare facilities in the region. The staff of these public facilities may have understood and come to the realization from these trainings the importance of promptly and professionally informing patients of medicine stock-outs.

## Discussion of results

The study established that the majority of respondents (patients) in the UER attended public healthcare facilities. This finding confirms the results of a cross-sectional study by [38], which concluded that most of the Kenyan population is mainly served by government healthcare providers. The results also indicated that the majority of patients to the public healthcare facilities did not receive medical commodities during their visits. This finding is in line with those found in Ghana by [6, 19] who established that patients were unable to receive all their entitled medical commodities at a public healthcare facility but ended up collecting prescription forms to buy these drugs from private drug stores and pharmacies [19] established that medical commodities, such as anti-malaria drugs, contraceptives and vaccines often have the most stock-outs, especially in public healthcare facilities. Theoretically, these findings are in sync with Andersen’s theory of behavioural use of health care facilities as it was established that the usage of public and private health care facilities in the region was influenced by the availability of medical commodities, frequency of medicine stock-outs, seasonality factors on medicine stock-outs, patients reaction to medicine stock-outs and communication to patients about medicine stock-outs. Hence medical commodity availability generally influences patients’ use of health care facilities in the UER of Ghana.

This finding concurs with the study outcomes by [23], who established that the management of malaria drugs in Zambia may be seasonally driven [11] established that Ghana’s annual rainy season commenced in May until September, with a substantial increase in the demand for anti-malaria drugs during this period.

The study also established that the majority of respondents of both private and public healthcare facilities did not always receive all their required essential medical commodities when attending a hospital. These results are similar to those found by [11, 23], who found low medical commodity availability at last mile public clinics was a major public health problem in SSA. [18] identified ineffective inventory controls, insufficient safety stock levels and poor record-keeping as notable causes of medicine stock-outs in SSA. This finding is not surprising, since private healthcare facilities can freely procure commodities from private suppliers. In contrast, according to a Ghana health service policy, public healthcare facilities may only procure commodities after a certificate of non-availability has been issued by the regional medical stores [12]. These findings are in line with studies conducted by [11].

Except for communication to patients about medicine stock-outs, where there was a significantly large difference, the t-test results in Table 11 showed small differences in the availability of commodities (AMC), frequency of medicine stock-outs (FMS), the effect of seasonality on medicine stock-outs (SMS) and the reactions to medicine stock-outs (RCN) of the private and public healthcare facilities. This means the communication about the medicine stock-outs was comparatively better in public healthcare facilities than the private facilities. This confirms a similar outcome of a comparative study by [34] among the Nepalese public and private healthcare facilities and the same was found in the UER of Ghana.

**Table 11:** Differences in patients’ perceptions of the factors influencing the delivery of medical commodities between private and public facilities

	**Private facility**	**Public facility**	**Levene’s test of equal variances**	**T-test for equal means**
	*n (Mean)(SD)*	*n (Mean)(SD)*	*p-value*	*p-value*
Availability of medical commodities	750 (2.99)(0.95)	750 (2.73)(1.01)	.206	**.000**
Frequency of medicine stock-outs	725 (3.11)(0.81)	724 (3.27)(0.82)	.352	**.000**
Seasonality factors on medicine stock-outs	725 (2.90)(0.80)	724 (3.11)(0.75)	**.002**	**.000**
Reaction to medicine stock-outs	725 (1.71)(0.43)	724 (1.78)(0.62)	**.000**	**.006**
Communication about the medicine stock-out	691 (2.60)(0.96)	660 (3.24)(0.98)	.371	**.000**

n = Sample size, *SD*  Standard deviation

Source: Author’s own compilation (2021)

Since the overall objective of the medical commodity supply chain is to ensure patients receive good quality medical commodities anytime they call in sick, managers of these commodity supply chains in the UER have to explore new ways to allow public healthcare facilities to procure medicines directly from private sources when medicine stock-outs occur. To improve medical commodity availability along the commodity supply chain in the region, the researcher strongly recommends that the managers of the public commodity supply chains apply for the revision of the public procurement guidelines on medicines. This would be to allow for direct procurement from private sources when the need arises, since the frequency of medicine stock-outs is comparatively higher in public than in private healthcare facilities.

This study found significant differences between the perceptions of patients of private and public healthcare facilities in respect of the availability of commodities, the frequency of medicine stock-outs, the effect of seasonality factors on medicine stock-outs, the reaction to medicine stock-outs, and communication about the medicine stock-outs. These differences were small for all factors except for patients’ communication about medicine stock-outs at private and public healthcare facilities, where the difference was large. Since the study concluded that better communication about the medicine stock-outs existed in private healthcare facilities compared to those in the public healthcare facilities, senior managers of the public commodity supply chain ought to redouble their efforts to improve this situation.

## Conclusions, limitations and future studies

This study was based on the views of patients who accessed medical commodities either from private or public healthcare facilities in the Upper East Region of Ghana. To generalise these findings, future studies should investigate the medical commodity supply chains of other regions of the country which may have different demographic, infrastructural, socio-economic or cosmopolitan characteristics compared to this region.

The data for this study were collected in 2018 implying that any developments since then to improve the efficiency of the supply of medical commodities in the region may not have been captured, especially with the advent of the COVID-19 pandemic. Despite the possibility of new developments, no new literature on the topic currently exists, making the results of this study still relevant. Finally, the findings of this study were based on cross-sectional data. The use of longitudinal data may provide broader insights into the operations of the medical commodity supply chains in the context of the UER.

## Data Availability

The datasets used and/or analysed during the current study available from the corresponding author on reasonable request.

## Footnotes

1 ETA squared of 0.01 implies small effect size; 0.06 is moderate and 0.14 implies large effect size (Cohen, 1988).

## References

[1] Africa Renewal. Health care systems: time for a rethink, United Nations: New York, USA. 2017. https://www.un-ilibrary.org/content/journals/25179829. Accessed 21 Oct 2020.

[2] Agarwal S, Tamrat T, Fønhus MS, Henschke N, Bergman H, Mehl GL, Glenton C, Lewin S (2018). Tracking health commodity inventory and notifying stock levels via mobile devices. Cochrane Database Syst Rev.

[3] Anabila P, Kumi D, Anome J (2019). Patients perceptions of healthcare quality in Ghana: a review of public and private hospitals. Int J Health Care Qual Assur.

[4] Andoh-Adjei F, Nsiah-Boateng E, Ankomah AF, Spaan E, Van der Velden K (2018). Perception of quality health care delivery under capitation payment: a cross-sectional survey of health insurance subscribers and providers in Ghana. BMC Fam Pract.

[5] Arney L, Yadav P. Improving procurement practices in developing country health programs, William Davidson Institute, University of Michigan, USA. 2014. https://wdi.umich.edu/wp-content/uploads/WDI-Improving-Procurement-Practice-in-Developing-Countries. Accessed 19 Nov 2020.

[6] Ashigbie P, Azameti D, Wirtz V (2016). Challenges of medicines management in the public and private sector under Ghana’s National Health Insurance Scheme–A qualitative study. J Pharm Policy Pract.

[7] Boakye K, Blankson C, Prybutok V, Qin H (2017). An assessment of national health care service delivery: a Ghanaian illustration. Int J Qual Reliab Manag.

[8] Chen C, Gu D. Andersen Model. In: Gu D, Dupre M.E, editors. Encyclopedia of gerontology and population aging. Cham: Springer; 2021. 10.1007/978-3-319-69892-2_876-1.

[9] Cohen L, Manion L, Morrison K (2007). Research Methods in Education.

[10] Fenny PK, Asante FA, Enemark U, Hansen KS (2015). Malaria care seeking behavior of individuals in Ghana under the NHIS: Are we back to the use of informal care?. BMC Public Health.

[11] Frimpong G, Ofori-Kwakye K (2016). Access to essential medicines in Ghana: a survey of availability of children’s medicines in medicine outlets in the Ashanti Region. J Appl Pharm Sci.

[12] Ghana Health Service (GHS). Private health sector development policies, Ministry of Health. Accra Ghana. 2004. www.ghanahealthservice.org/downloads/Ghana_Health_Service_2004_Annual_Report.pd. Accessed on 11 Apr 2020.

[13] Ghana Health Service (GHS). The Ghana weekly epidemiological report, Ghana Health Service, Accra - Ghana. 2017. www.ghanahealthservice.org/downloads/Ghana_Health_Service_2017_Annual_Report.pdf. Accessed on 18 Sept 2020.

[14] Ghana Health Service. Ghana Health Services: The health sector in Ghana: Facts and Figures, Accra-Ghana. 2018. Available from: https://www.ghanahealthservice.org/downloads/Ghana_Health_Service_2018_Annual_Report.pdf. Accessed on 2 Nov 2020.

[15] Ghana Statistical Service. (GSS). The 2021 Housing and Population census report, Ghana Statistical Service, Accra. 2021. https://www.ghanastatisticalservice.org/downloads/Ghana_Statistical_Service_2021_Annual_Report.pdf. Accessed on 18 Dec 2022.

[16] Institute of Health Metrics and Evaluation (IHME). Findings from the Global Burden of Disease Study:An annual report, Seattle, WA. 2018. Available from:https://www.healthdata.org/sites/default/files/files/policy_report/2013/GBD_GeneratingEvidence/IHME. Accessed on 5 June 2021.

[17] Kabir MR (2021). Adopting Andersen’s behavior model to identify factors influencing maternal healthcare service utilization in Bangladesh. PLoS One.

[18] Kefale A, Hayredin H, Shebo T (2019). Availability of essential medicines and pharmaceutical inventory management practice at health centers of Adama town Ethiopia. BMC Health Serv Res.

[19] Kotoh AM, Aryeetey GC, Van der Geest S (2018). Factors that influence enrolment and retention in Ghana’s National Health Insurance Scheme. Int J Health Policy Manag.

[20] Kushitor M, Boatemaa S (2018). The double burden of disease and the challenge of health access: evidence from access, bottlenecks, cost and equity facility survey in Ghana. PLoS One.

[21] Kwateng CA, Iyer-Raniga U, Guillermo U (2018). Transport and accessibility challenges facing the rural people living along feeder roads in Ghana. Civ Eng Arch.

[22] Laar AK, Kwara A, Nortey PA, Ankomah AK, Okyerefo MPK, Lartey MY (2017). Use of non-prescription remedies by Ghanaian human immunodeficiency virus-positive persons on antiretroviral therapy. Public Health.

[23] Leung N-HZ, Chen A, Yadav P, Gallien J (2017). The impact of inventory management on stock-outs of essential drugs in sub-Saharan Africa: secondary analysis of a field experiment in Zambia. PLoS One.

[24] Martins RR, Farias AD, Oliveira YMC, Diniz RS, Oliveira AG (2017). Prevalence and risk factors of inadequate medicine home storage: a community-based study. Rev Saude Publica.

[25] Ministry of Local Government and Rural Development (MLGRD). Metropolitan, Municipal and Districts assemblies in Ghana, 2015. Annual report. the ministry of local government and rural development Accra-Ghana. 2019. http://www.mlgrd.gov.gh/library/document. Accessed on 17 Nov 2019.

[26] Naazie AN, Braimah SR, Atindana VA (2018). The effects of bad roads on transportation systems in the Gushegu district of Northern Region of Ghana. Am Sci Res J Eng Technol Sci.

[27] Nonvignon J, Aryeetey GC, Malm KL, Agyemang SA, Aubyn VNA, Peprah NY, Bart-Plange CN, Ainkins M (2016). Economic burden of malaria of businesses in Ghana: a case for private sector investment in malaria control. Malar J.

[28] Nuche-Berenguer B, Kupfer L (2018). Review article readiness of sub Saharan Africa healthcare systems for the new pandemic, diabetes: a systematic review. Hindawi J Diabetes Research.

[29] Olanrewaju S, Onyekachi M (2020). Comparison of principal component analysis, maximum likelihood and the principal axis in factor analysis. Am J Math Stat.

[30] Pallant J. A step-by-step guide to data analysis using SPSS. 4th Edition. 2016. Available from:www.cal.org/twi/EvalToolkit/appendix/toolkit13_sec9.pdf. Accessed on 7 Apr 2018.

[31] Personal Action Towards Health (PATH). Diabetes supplies: Are they there when needed? A PATH Report, Seattle, U.S.A. 2015. www.path.azureedge.net/media/documents/NCD_nes_exec_summary.pdf. Accessed on 2 Jan 2018.

[32] Saunders M, Lewis P, Thornhill A (2016). Research methods for business students.

[33] Tesfaye G, Chojenta C, Smith R, Loxton D (2018). Application of the Andersen-Newman model of health care utilization to understand antenatal care use in Kersa District Eastern Ethiopia. PLoS One.

[34] Tiwary A, Rimal A, Paudyal B, Sigdel R, Basnyat B (2019). Poor communication by health care professionals may lead to life-threatening complications: examples from two case reports. Wellcome Open Res.

[35] Tolera H, Gebre-Egziabher T, Kloos H (2020). Using Andersen’s behavioral model of health care utilization in a decentralized program to examine the use of antenatal care in rural western Ethiopia. PLoS One.

[36] Tynkkynen L, Vrangbæk G (2018). Comparing public and private providers: a scoping review of hospital services in Europe. BMC Health Serv Res.

[37] Varela C, Young S, Mkandawire N, Groen RS, Banza L, Viste A (2019). Transportation barriers to access health care for surgical conditions in Malawi: a cross sectional nationwide household survey. BMC Public Health.

[38] Wambiya EO, Otieno P, Mutua K, Pythagore H, Donfouet P, Mohamed S (2021). Patterns and predictors of private and public health care utilization among residents of an informal settlement in Nairobi, Kenya: a cross-sectional study. BMC Public Health.

[39] Wang W, Maitland E, Nicholas S (2017). Comparison of patient perceived primary care quality in public clinics, public hospitals and private clinics in rural China. Int J Equity Health.

[40] Wang C, Cao H (2019). Persisting Regional disparities in modern contraceptive use and unmet need for contraception among Nigerian women. Hindawi BioMed Res Int.

[41] World Health Organisation (WHO). WHO library cataloguing-in-publication data: World health statistics 2015, The World Health Organisation, Geneva - Switzerland. 2018. http://www.who.int/gho/publications/world_health_statistics/EN_WHS2015_TOC.pdf. Accessed 12 Jan 2020.

[42] World Health Organisation (WHO). Combatting the disease burden in sub-Saharan, Africa World Health Organisation. Geneva, Switzerland. 2019. http://www.who.int/bulletin/archives/79(10)947.pdf?ua=1. Accessed on 12 Jan 2020.

[43] World Bank. Infrastructure development in sub-Saharan Africa: a scorecard, policy research working paper 8425. IBRD, Washington DC. 2018. http://openknowledge.worldbank.org/bitstream/handle/10986/29770/WPS8425.pdf. Accessed on 23 Mar 2018.

